# Genome-wide association mapping of seed oligosaccharides in chickpea

**DOI:** 10.3389/fpls.2022.1024543

**Published:** 2022-10-24

**Authors:** Dinakaran Elango, Wanyan Wang, Mahender Thudi, Sheelamary Sebastiar, Bharathi Raja Ramadoss, Rajeev K. Varshney

**Affiliations:** ^1^ Department of Agronomy, Iowa State University, Ames, IA, United States; ^2^ Department of Plant Science, Penn State University, University Park, PA, United States; ^3^ Ecosystem Science and Management, Penn State University, University Park, PA, United States; ^4^ Department of Agricultural Biotechnology and Molecular Biology, Dr. Rajendra Prasad Central Agricultural University, Samastipur, India; ^5^ Centre for Crop Health, University of Southern Queensland (USQ), Toowoomba, QLD, Australia; ^6^ Genetics Gains Research Program, International Crops Research Institute for the Semi-Arid Tropics (ICRISAT), Hyderabad, India; ^7^ Division of Crop Improvement, Indian Council of Agricultural Research (ICAR)-Sugarcane Breeding Institute, Coimbatore, India; ^8^ Saskatoon Research and Development Centre, Agriculture and Agri-Food Canada, Saskatoon, SK, Canada; ^9^ State Agricultural Biotechnology Centre, Crop Research Innovation Centre, Food Futures Institute, Murdoch University, Murdoch, WA, Australia

**Keywords:** anti-nutritional factors (ANF), flatus potential, marker trait associations, prebiotics, raffinose family oligosaccharides (RFOs), specialty chickpeas

## Abstract

Chickpea (*Cicer arietinum* L.) is one of the major pulse crops, rich in protein, and widely consumed all over the world. Most legumes, including chickpeas, possess noticeable amounts of raffinose family oligosaccharides (RFOs) in their seeds. RFOs are seed oligosaccharides abundant in nature, which are non-digestible by humans and animals and cause flatulence and severe abdominal discomforts. So, this study aims to identify genetic factors associated with seed oligosaccharides in chickpea using the mini-core panel. We have quantified the RFOs (raffinose and stachyose), ciceritol, and sucrose contents in chickpea using high-performance liquid chromatography. A wide range of variations for the seed oligosaccharides was observed between the accessions: 0.16 to 15.13 mg g^-1^ raffinose, 2.77 to 59.43 mg g^-1^ stachyose, 4.36 to 90.65 mg g^-1^ ciceritol, and 3.57 to 54.12 mg g^-1^ for sucrose. Kabuli types showed desirable sugar profiles with high sucrose, whereas desi types had high concentrations RFOs. In total, 48 single nucleotide polymorphisms (SNPs) were identified for all the targeted sugar types, and nine genes (*Ca_06204, Ca_04353*, and *Ca_20828*: *Phosphatidylinositol N-acetylglucosaminyltransferase*; *Ca_17399* and *Ca_22050*: *Remorin proteins*; *Ca_11152*: *Protein-serine/threonine phosphatase; Ca_10185, Ca_14209*, and *Ca_27229*: *UDP-glucose dehydrogenase*) were identified as potential candidate genes for sugar metabolism and transport in chickpea. The accessions with low RFOs and high sucrose contents may be utilized in breeding specialty chickpeas. The identified candidate genes could be exploited in marker-assisted breeding, genomic selection, and genetic engineering to improve the sugar profiles in legumes and other crop species.

## Introduction

Chickpea (*Cicer arietinum* L.) is one of the founder crops domesticated between 9,000–11,000 years ago and is an important ancient legume grown and consumed all over the globe (Lev-Yadun et al., 2000). As a legume crop, it is often grown as rotational crops with cereals to enhance yield because of their ability to fixing atmospheric nitrogen (Graham and Vance, 2003). Chickpea is rich in carbohydrates (60-65%), protein (20-22%), fat (6%), and rich in dietary fiber, as well as minerals (phosphorus, calcium, magnesium, iron, and zinc) and vitamins (β-carotene, thiamin, riboflavin, and niacin) (Jukanti et al., 2012). The major pulse grain constituents are carbohydrates, based on their polymeric structure that can be classified as monosaccharides (ribose, fructose, and glucose), disaccharides (sucrose, maltose, melibiose), oligosaccharides (raffinose, stachyose, verbascose, ajugose, and ciceritol) and polysaccharides (Chibbar et al., 2010). Among the oligosaccharides, *α-galacto-oligosaccharides* (*α-GOS*) are known as raffinose family oligosaccharides (RFOs) (Sosulski et al., 1982). The major RFOs found in chickpea include raffinose, stachyose, and verbascose. However, ciceritol does not belong to the RFOs since its structure is different from *α-GOS* and can rapidly undergo a hydrolysis process, so unlike raffinose and stachyose, ciceritol does not cause flatulence in humans and animals (Quemener and Brillouet, 1983).

RFOs are the single most deterrent factor for the rapid adoption of legumes in mainstream food usage in humans and animals (Delumen, 1992; Elango et al., 2022a). Humans and animals lack the enzyme *α-galactosidase* to degrade *α-galactosides* (RFOs), which results in the accumulation of undigested RFOs in the large intestine of the digestive system, which ultimately causes flatulence and abdominal discomforts due to the production of flatulent gases by colonic bacteria through fermenting the un-digested RFOs present in the guts (Calloway and Murphy, 1968; Sosulski et al., 1982; Singh, 1985; Han and Baik, 2006). Though, RFOs have been reported to have a beneficial effect on gut microflora (Van den Ende, 2013; Su et al., 2019), and play a role in seed germinability and biotic and abiotic stress tolerance in crop plants (Gulewicz et al., 2002; Taji et al., 2002; Nishizawa-Yokoi et al., 2008; Dobrenel et al., 2013; Van den Ende, 2013; Gangl and Tenhaken, 2016; Yan et al., 2022). However, we do not know what the right concentration is needed to benefit humans, animals, and plants concerning RFOs.

Food processing can eliminate RFOs at varying degrees and significantly increase dietary fraction availability in food (Jood et al., 1985; Egbe and Akinyele, 1990; Aguilera et al., 2009). However, these techniques often come with trade-offs; most techniques are time-consuming, lead to loss of nutrients, and sometimes have consumer acceptability issues. Therefore, identifying sources of variation for developing desirable sugar-type cultivars through crop breeding is very important. Screening and identification of low RFOs have been carried out in many economically important legume crops such as lentil (Tahir et al., 2011), chickpea (Raja et al., 2015; Gangola et al., 2016), pea (Peterbauer et al., 2003), soybean (Blackman et al., 1992; Dierking and Bilyeu, 2008; Obendorf and Górecki, 2012), mung bean and urd bean (Souframanien et al., 2014), whereas, very limited efforts have been taken toward the identification of genomic regions associated with RFOs in crop plants. In this context, our study aims to identify the genetic factors responsible for seed oligosaccharides in chickpea through genome-wide association mapping using the International Crops Research Institute for the Semi-Arid Tropics (ICRISAT) mini-core collection.

## Materials and methods

### Plant materials

The chickpea mini-core collection consisting of 211 accessions from 24 countries (Asia, Africa, Europe, North, and South American regions) was obtained from the genetic resources division of International Crops Research Institute for the Semi-Arid Tropics (ICRISAT), India (Upadhyaya and Ortiz, 2001). Field experiments were performed in a randomized complete block design (RCBD) with three replications in the 2010 winter season at the Department of Pulses (11.0232° N latitude, 76.9293° E longitude, 426.72 m altitude), Tamil Nadu Agricultural University (TNAU), Coimbatore, India. Each accession was grown in a single row in a 3 m long plot. Seeds from each replicate of individual accessions were harvested at physiological maturity and stored at 4°C until analysis was performed. A standard agronomic package of practices was followed to achieve the best crop establishment.

### Sugar extraction and quantification

The seeds of each chickpea accession were grounded, and the flours were used to extract soluble sugars. One gram of flour samples was taken into a screw cap vial and mixed with 10 ml of 50% ethanol, and vortexed briefly. After adding ethanol, samples were shaken horizontally using a water bath shaker maintained at 50° C for 30 min at 100 rpm. The incubated vial was centrifuged at 4000 rpm for 5 min, then 5 ml of supernatant was taken and mixed with 7 ml of acetonitrile (high-performance liquid chromatography (HPLC)-grade) to precipitate the soluble proteins. The mixture (5 ml supernatant + 7 ml acetonitrile) was incubated at room temperature for two hours. After incubation, the mixture was centrifuged at 3670 g for 5 min, and one ml aliquot of the supernatant was collected. The collected supernatant was dried at 50°C and resuspended with 500 μl 65% HPLC-grade acetonitrile and filtered through a 0.2 μm membrane filter and transferred to HPLC vials. Standards of sucrose, raffinose, and stachyose were purchased from Sigma-Aldrich, Bengaluru, India and ciceritol was purchased from Clearsynth, Hyderabad, India. Three different concentrations, 1.25 mg ml^-1^, 2.5 mg ml^-1^, and 5.0 mg ml^-1^, were prepared and included in each batch of samples to obtain the standard curve. The concentration of different sugars (sucrose, raffinose, stachyose, and ciceritol) was determined using the HPLC (Shimadzu, Kyoto, Japan), which consisted of an LC20AD pump and a RID-10A refraction index detector. Sugar concentrations were determined using the peak area of the sample in comparison with standards.

### Marker-trait association analyses

We performed genome-wide association mapping analysis using 673,115 single nucleotide polymorphisms (SNPs), where the SNP calls for 211 genotypes were obtained from Varshney et al. (2019). As reported in Varshney et al. (2019), for calling SNPs, the clean reads were mapped on to the reference genome of chickpea genotype CDC Frontier using SOAP2. We then used SOAPsnp3 to calculate the likelihood of all possible genotypes for each sample. In order to filter out low-quality variants, the loci with sequencing depth higher than 10,000 and lower than 400, mapping times higher than 1.5, or quality scores lower than 20, were filtered out. The loci with estimated allele frequency not equal to 0 or 1 were determined as SNPs. After obtaining the SNPs, we also determined the genotype of each individual at the SNP locus by assigning the most likely genotype from the SOAPsnp3 result of each sample. We have used the Fixed and random model Circulating Probability Unification (FarmCPU) model (Liu et al., 2016) in the Genome Association and Prediction Integrated Tool (GAPIT3) package (Wang and Zhang, 2021) to identify significant marker-trait associations (MTAs). GAPIT estimated the allelic effect for the significant SNPs identified. Sign (+/-) of the allelic effect estimate is relative to the alphabetical order of the nucleotides. MTAs were selected for *p*-value <10^-5^. Gene annotations were determined from the reference genome of chickpeas released in 2013 (Varshney et al., 2013). Genes within the flanking regions of 50kb upstream and downstream of the significantly called SNPs were collected first, and among them, only the genes already annotated in the chickpea genome with predicted function or found orthologs in other model plants were selected as candidate genes. Distance from the SNP (bp) is calculated as the distance from the SNP location to the start site of the upstream or downstream genes. If the SNP is located within a gene, the distance is calculated as the distance to the start site of the gene.

## Results

### Phenotypic variation and correlations among seed oligosaccharides

**Figure 1:**
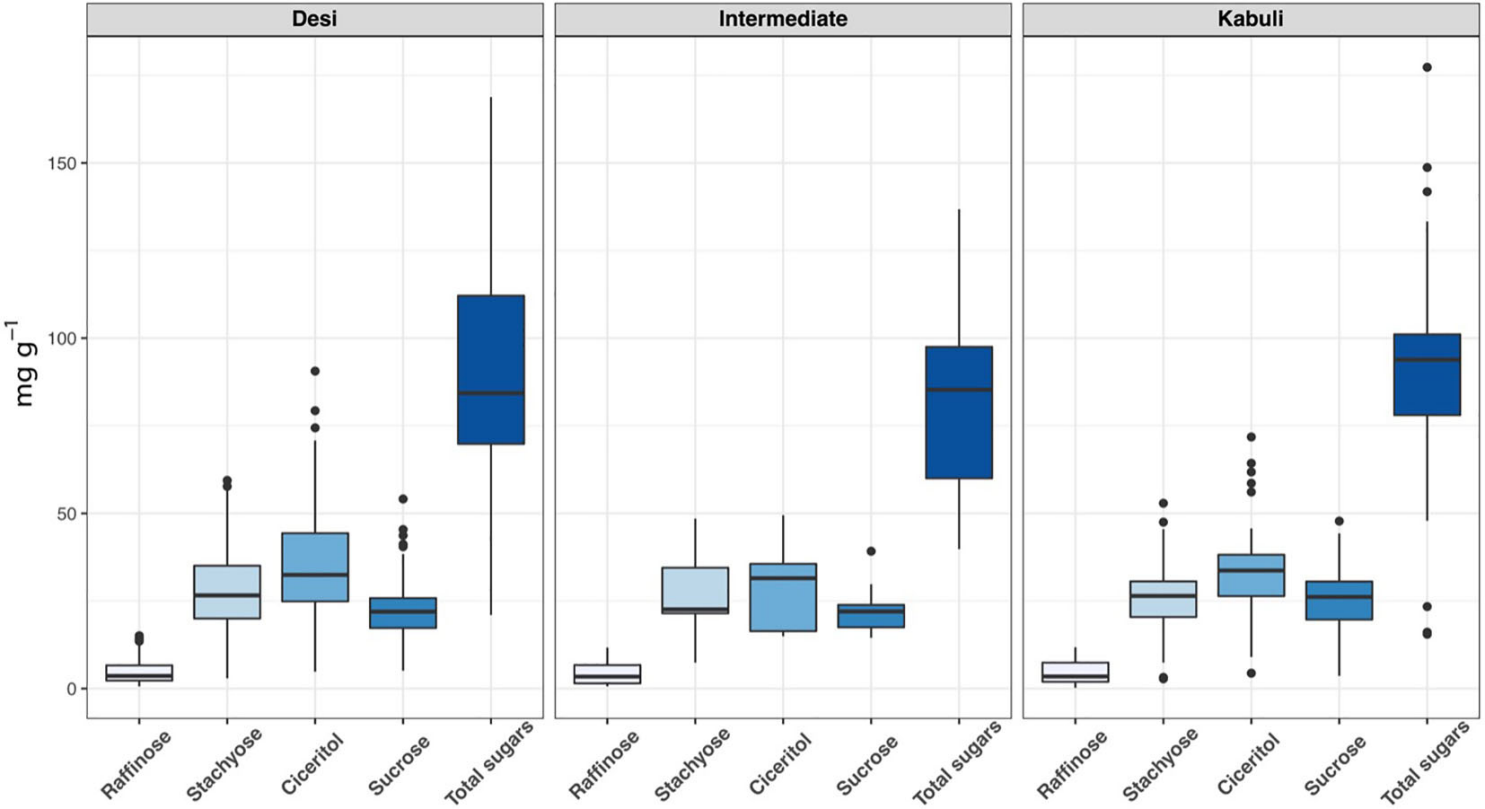
Seed oligisaccharides variations among desi, kabuli, and intermediate types in the ICRISAT chickpea mini-core panel.

**Figure 2:**
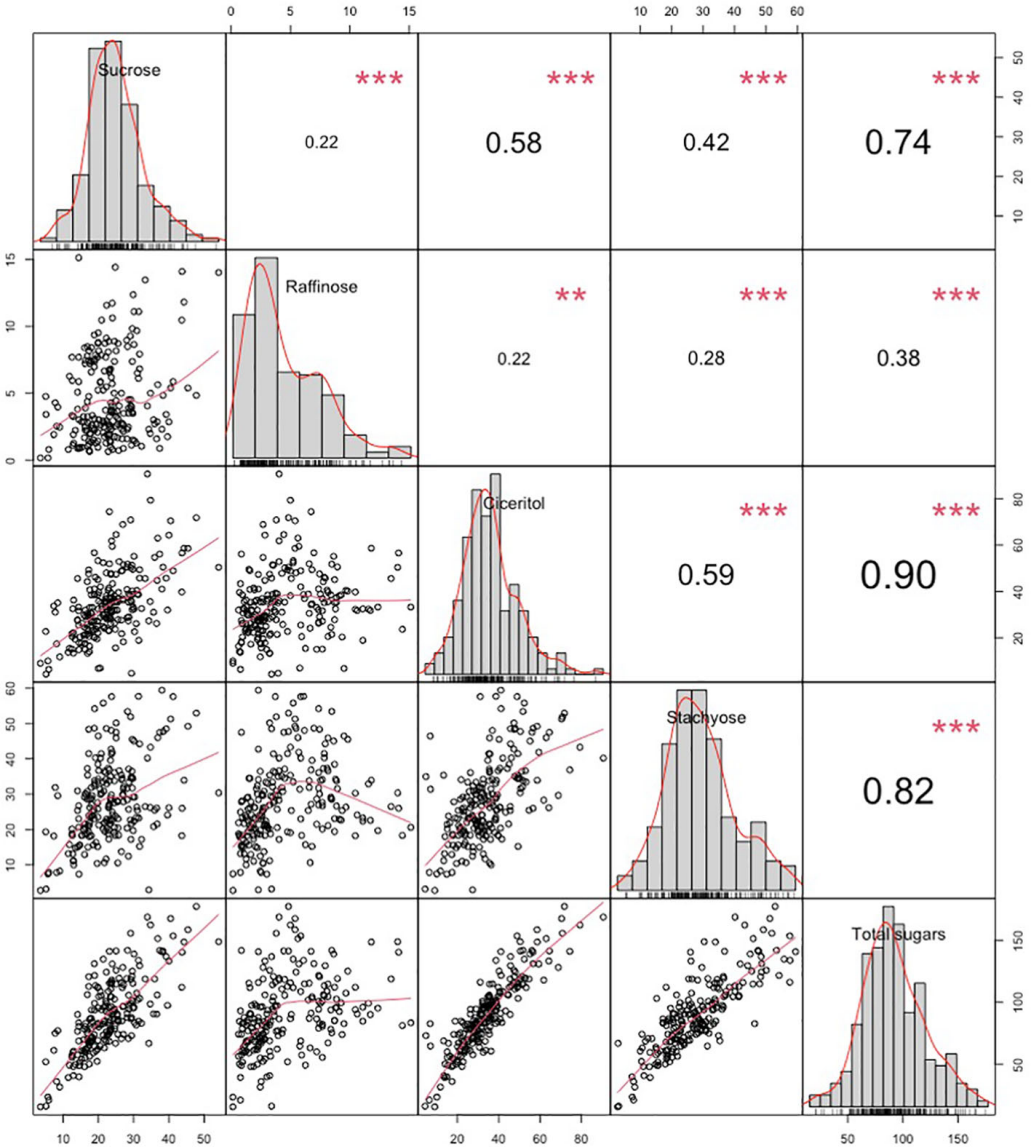
Variation and Pearson pairwise correlations of sucrose, raffinose, ciceritol, stachyose, and total sugars in chickpea. Upper diagonal: Pearson correlation coefficients between every two traits. Mid-diagonal: Histograms of sucrose, raffinose, ciceritol, stachyose, and total sugars. Lower diagonal: Bivariate scatter plots of correlations between every two traits with a fitted line. **Significant at the .01 probability level. ***Significant at the .001 probability level.

We have observed wide variations for all sugars measured in the ICRISAT chickpea mini-core collection. Among the morphotypes, Kabuli-type chickpeas exhibited higher sucrose and total sugar contents. In contrast, the desi-type chickpeas showed higher RFOs (raffinose and stachyose) and ciceritol contents in seeds (**Figure 1**). Mini-core collection showed wide seed oligosaccharide variations: 0.16 to 15.13 mg g^-1^, 2.77 to 59.43 mg g^-1^, 4.36 to 90.65 mg g^-1^, 3.57 to 54.12 mg g^-1^ for raffinose, stachyose, ciceritol, and sucrose with an average of 4.61, 28.02, 34.48, and 23.11 mg g^-1^ flour sample, respectively (**Table 1**). We have observed significant positive correlations among seed oligosaccharides measured: sucrose, ciceritol, and stachyose have strong positive correlations with total sugars; moderate correlations were observed between sucrose and ciceritol, and ciceritol and stachyose (**Figure 2**). Whereas raffinose had a low level of positive correlations with all other seed oligosaccharides (**Figure 2**).

**Table 1:** Variability of chickpea accessions for seed oligosaccharide contents.

Seed oligosaccharides	Range	Mean ± SD	Desirable sugar profile genotypes	Origin	Morphotype
	mg g^-1^				
Raffinose	0.16 - 15.13	4.61 ± 3.24	ICC 6263 (0.16)	Russia & CISs	Kabuli
Stachyose	2.77 - 59.43	28.02 ± 11.69	ICC 13816 (2.77)	Russia & CISs	Kabuli
Ciceritol	4.36 - 90.65	34.48 ± 14.38	ICC 12968 (4.36)	India	Kabuli
Sucrose	3.57 - 54.12	23.11 ± 8.32	ICC 12654 (54.12)	Ethiopia	Desi
Total sugars	15.53 - 177.34	90.22 ± 29.93	–	–	–

### Marker trait associations

**Figure 3:**
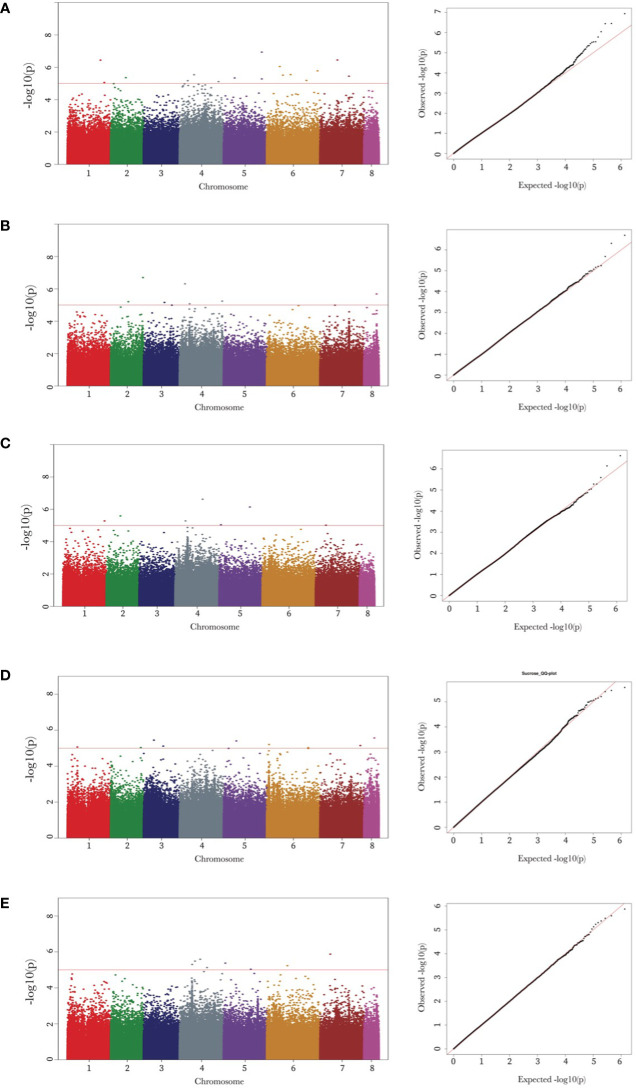
Manhattan and quantile-quantile **(Q-Q)** plots of raffinose, stachyose, ciceritol, sucrose, and total sugars in chickpea. Manhattan and Q-Q plots of the seed oligosaccharides from a to e are as follows: raffinose **(A)**, stachyose **(B)**, ciceritol **(C)**, sucrose **(D)**, and total sugars **(E)**. Negative log_10_ transformed *P* values (y-axis) are plotted against the physical single nucleotide polymorphism (SNP) position on each chromosome (x-axis). Each circle represents a SNP, and the corresponding SNPs were mentioned trait wise. The dotted red line represents the genome-wide significance threshold as determined by Bonferroni correction at.05. Regions with negative log_10_ *P* values above the threshold contain quantitative trait loci candidates.

Genome-wide association mapping identified 48 SNPs associated with the seed oligosaccharide contents in chickpeas (**Table 2**). The largest number of associated markers (12) were detected on chromosome 4 (**Table 2**), and there were 16 SNPs found to be significantly associated with raffinose content in chickpea, which is the most associated markers compared with other three sugars: 7 SNPs for ciceritol and stachyose respectively, and 9 SNPs for sucrose, and 8 SNPs for total sugar content in chickpea (**Table 2**; **Figure 3**).

**Table 2:** List of significant single nucleotide polymorphic associations, the genes tagged by significant single nucleotide polymorphic markers, and candidate genes identified based on proximity to the significant markers and their description for ciceritol, raffinose, stachyose, sucrose, and total sugars in chickpea.

Oligosaccharides	Scaffold position	Allele	*P* value	Minor allele frequency	Allelic effect	Chickpea gene ID	Distance from the SNP (bp)*	SNP position to gene loci	Functional annotation
Ciceritol	***Ca4_30621427***	***A/T***	***2.38E-07***	***0.26***	***-5.54***	***Ca_14209***	***8067***	***Downstream***	***UDP-glucose dehydrogenase***
						Ca_14210	47995	Upstream	TBCC domain-containing protein 1
	Ca5_34185294	C/A	7.22E-07	0.24	7.07	Ca_01829	5411	Downstream	Cryptochrome, DASH family protein
						Ca_01828	1764	Upstream	Predicted membrane protein
	Ca2_15589436	G/C	2.57E-06	0.47	13.91	Ca_18544	47000	Downstream	6-phosphogluconate dehydrogenase
						Ca_18543	19728	Upstream	Receptor-like protein kinase-related
	Ca4_11536839	A/G	5.22E-06	0.43	-6.05	Ca_04384	46920	Downstream	Tubby-like f-box protein 1-related
						Ca_04385	15105	Upstream	Trihelix transcription factor gt-2
	Ca1_46225454	T/C	5.24E-06	0.06	-9.08	Ca_21541	3116	Within the gene	Tpx2 (targeting protein for xklp2) protein family
	Ca5_1870839	G/A	9.02E-06	0.17	-8.41	Ca_26715	68	Within the gene	DNA damage-binding protein 1 (DDB1)
	Ca7_11316700	A/G	9.64E-06	0.28	-6.74	Ca_09363	20294	Downstream	AhpC/TSA antioxidant enzyme (AhpC-TSA_2)
						Ca_09362	7497	Upstream	CAMP-response element binding protein-related
Raffinose	Ca5_42575153	T/A	1.16E-07	0.24	-1.99	Ca_11357	12497	Downstream	F-box only protein 6
						Ca_11356	6613	Upstream	Squamosa promoter-binding-like protein 10-related
	Ca7_19222249	T/C	3.54E-07	0.19	-1.43	Ca_12328	7908	Downstream	Ap2-like ethylene-responsive transcription factor ail6-related
						Ca_12329	6606	Upstream	Tetratricopeptide repeat
	***Ca1_36822662***	***G/A***	***3.61E-07***	***0.15***	***-2.30***	Ca_21700	29080	Downstream	Ulp1 protease family
						***Ca_27229***	***17117***	***Upstream***	***Udp-glucose dehydrogenase***
	Ca6_14474273	T/C	9.04E-07	0.12	-1.77	Ca_05217	5850	Downstream	Protein-serine/threonine phosphatase
						Ca_05218	16903	Upstream	Cotton fibre expressed protein
	***Ca6_56636684***	***T/A***	***1.66E-06***	***0.12***	***-2.37***	***Ca_17399***	***1462***	***Downstream***	***Remorin family protein***
						Ca_17400	1515	Upstream	RNAse P Rpr2/Rpp21/SNM1 subunit domain (Rpr2)
	Ca6_26580794	T/C	2.85E-06	0.25	-1.33	Ca_16690	13777	Downstream	Dof domain, zinc finger (zf-Dof)
						Ca_16691	18135	Upstream	WUSCHEL-related homeobox 2
	Ca4_16178393	G/A	2.89E-06	0.16	-1.59	Ca_05494	6388	Downstream	Zinc finger protein jagged-related
						Ca_05493	4880	Upstream	Protein istr-1, isoform a
	Ca6_18000446	T/G	3.06E-06	0.37	-2.07	Ca_06421	12470	Downstream	Serine/threonine-protein kinase srk2e
						Ca_06422	25071	Upstream	Myb family transcription factor
	Ca7_32073468	C/A	3.55E-06	0.13	-1.75	Ca_10064	24292	Downstream	NB-ARC domain (NB-ARC)/Leucine Rich Repeat (LRR_3)
						Ca_10063	2431	Upstream	Phospholipid-transporting ATPase tat-1
	***Ca2_16716210***	***T/C***	***4.40E-06***	***0.12***	***-1.60***	Ca_22049	1280	Downstream	Protein-serine/threonine phosphatase
						***Ca_22050***	***13224***	***Upstream***	***Remorin family protein***
	Ca5_12255667	T/C	4.58E-06	0.21	-1.34	Ca_17088	9464	Downstream	Anion exchange protein
						Ca_17087	3021	Upstream	Myb-like DNA-binding protein
	Ca5_42575149	T/A	5.24E-06	0.19	-1.93	Ca_11357	12493	Downstream	F-box only protein 6
						Ca_11356	6617	Upstream	Squamosa promoter-binding-like protein 10-related
	Ca6_44267477	T/C	6.50E-06	0.05	-2.52	Ca_24232	23675	Downstream	Aldo-keto reductase family
						Ca_24233	62720	Upstream	Transposon protein, putative, CACTA, En/Spm sub-class
	Ca4_9041839	G/C	6.76E-06	0.20	-1.86	Ca_08396	21870	Downstream	Thioredoxin-like protein
						Ca_08397	112	upstream	Geraniol 8-hydroxylase
	Ca4_43438450	G/A	7.70E-06	0.06	-2.06	Ca_23689	97	Within the gene	Pollen proteins Ole e I like
	Ca1_41085856	T/A	8.75E-06	0.15	-1.91	Ca_18474	11499	Downstream	Disease resistance protein rpp13-related
						Ca_18475	9169	Upstream	Transposon protein, putative, CACTA, En/Spm sub-class
Stachyose	Ca2_35864450	G/A	2.00E-07	0.12	-7.18	Ca_09762	5676	Within the gene	Armadillo repeat-containing protein 8
	Ca4_6044239	C/T	4.87E-07	0.29	5.04	Ca_03642	543	Within the gene	Syntaxin 16
	Ca8_14016267	C/A	2.07E-06	0.09	-8.60	Ca_22742	9720	Downstream	RNA-binding protein
						Ca_22743	50107	Upstream	Mza15-related
	Ca4_47554909	T/G	5.72E-06	0.10	-7.21	Ca_10834	21050	Downstream	Uncharacterized conserved protein
						Ca_10833	7624	Upstream	Histone deacetylase complex
	Ca2_19532676	T/C	6.21E-06	0.07	-7.19	Ca_25228	65339	Downstream	N-acyl-aliphatic-L-amino acid amidohydrolase
						Ca_24535	57346	Upstream	Ulp1 protease family, C-terminal catalytic domain
	***Ca3_23073125***	***T/C***	***6.86E-06***	***0.14***	***-5.57***	***Ca_06204***	***14903***	***Downstream***	***Phosphatidylinositol n-acetylglucosaminyltransferase subunit p-like protein***
						Ca_06203	7050	Upstream	U3 small nucleolar RNA-associated protein 20
	Ca4_11202747	T/A	8.39E-06	0.14	-6.06	Ca_04352	12894	Downstream	Small subunit ribosomal protein S1
						***Ca_04353***	***7468***	***Upstream***	***Type I inositol 1,4,5-trisphosphate 5-phosphatase 1***
Sucrose	Ca8_11491206	C/A	2.70E-06	0.35	-3.97	Ca_16815	6142	Downstream	Transposon protein, CACTA, En/Spm sub-class, expressed
						Ca_16814	6618	Upstream	Prostaglandin-E synthase
	Ca3_11428639	G/A	3.56E-06	0.20	-5.45	Ca_19377	35461	Downstream	Aquaporin tip1-3
						Ca_19378	133058	Upstream	Two-component sensor histidine kinase
	***Ca5_14173812***	***G/A***	***3.96E-06***	***0.11***	***-5.06***	***Ca_20828***	***30851***	***Downstream***	***Multiple inositol-polyphosphate phosphatase/2,3-bisphosphoglycerate 3-phosphatase***
						Ca_20829	30444	Upstream	Calmodulin-binding protein
	Ca6_2510863	G/A	6.24E-06	0.06	-5.58	Ca_10383	2361	Within the gene	FI03258P
	Ca7_44816569	T/A	7.12E-06	0.10	5.91	Ca_15705	21617	Downstream	Zinc knuckle (zf-CCHC)
						Ca_15706	24202	Upstream	Camp-response element binding protein-related
	Ca3_21765752	C/T	7.67E-06	0.08	5.43	Ca_09529	17032	Downstream	Ethanolamine-phosphate cytidylyltransferase
						Ca_09530	8554	Upstream	GRAS family transcription factor
	Ca1_10947431	T/C	8.62E-06	0.22	3.13	Ca_02666	9994	Downstream	WRKY transcription factor 65-related
						Ca_02665	4707	Upstream	Cyclin-B1-4
	***Ca2_33431205***	***T/A***	***9.25E-06***	***0.06***	***-6.88***	***Ca_10185***	***2477***	***Downstream***	***Udp-glucose dehydrogenase***
						Ca_10184	44823	Upstream	Lipase containing protein
	Ca6_46242693	T/A	9.41E-06	0.19	-3.29	Ca_13883	34784	Downstream	Unknown
						Ca_13884	20164	Upstream	Phospholipase A(2)/Phospholipase A2
Total Sugar	Ca7_11316700	A/G	1.34E-06	0.28	-15.00	Ca_09363	20294	Downstream	AhpC/TSA antioxidant enzyme (AhpC-TSA_2)
						Ca_09362	7497	Upstream	CAMP-response element binding protein-related
	Ca4_22983648	T/C	2.57E-06	0.38	18.76	Ca_14460	14194	Downstream	Myb/SANT-like DNA-binding domain
						Ca_14459	67448	Upstream	F26K24.5 protein
	Ca4_17208915	C/T	3.26E-06	0.06	-19.82	Ca_05402	453	Within the gene	V-type H+-transporting ATPase subunit B
	Ca5_1870839	G/A	4.21E-06	0.17	-17.79	Ca_26715	68	Within the gene	DNA damage-binding protein 1
	Ca4_14043315	G/A	4.99E-06	0.37	10.93	Ca_04629	7392	Downstream	Homeobox-leucine zipper protein hdg2
						Ca_04630	2011	Upstream	MKIAA1688 protein
	***Ca6_22947128***	***A/T***	***5.86E-06***	***0.47***	***15.57***	Ca_11153	5645	Downstream	Peroxisomal targeting signal type 2 receptor
						***Ca_11152***	***4258***	***Upstream***	***Protein-serine/threonine phosphatase***
	***Ca4_30621427***	***A/T***	***7.43E-06***	***0.26***	***-9.89***	***Ca_14209***	***8067***	***Downstream***	***UDP-glucose dehydrogenase***
						Ca_14210	47995	Upstream	TBCC domain-containing protein 1
	Ca5_30294813	T/C	9.18E-06	0.36	-10.88	Ca_04706	2728	Downstream	Nipped-b-like protein delangin scc2-related
						Ca_04707	9082	Upstream	Aquaporin transporter

*Distance from the SNP (bp) is calculated as the distance from the SNP location to the start site of the upstream or downstream genes. If the SNP is located within a gene, the distance is calculated as the distance to the start site of the gene.

Probable candidate genes involved in seed oligosaccharides metabolism and transport are bolded and italicized.

### Probable candidate genes

We identified 80 probable candidate genes for all the seed oligosaccharides measured in this study (**Table 2**). Among them, nine genes were recognized with annotated functions highly associated with sugar biosynthesis and transportation. Four genes that are important components in inositol biosynthesis: two associated with stachyose (*Ca_06204: phosphatidylinositol N-acetylglucosaminyltransferase; Ca_04353: Type I inositol 1,4,5-trisphosphate 5-phosphatase*), and two associated with sucrose (*Ca_20828: inositol-polyphosphate phosphatase; Ca_10185*: *UDP-glucose dehydrogenase*) have been identified as probable candidates for seed oligosaccharide biosynthesis in chickpeas (**Table 2**). There were also three candidate genes identified playing an important role in RFOs transportation: two of the genes (*Ca_17399* and *Ca_22050*) are associated with raffinose content, and the other gene (*Ca_11152*) is linked with total sugar content in chickpea seeds, and all of them encodes *remorin protein*, which regulates the carbohydrate translocation in plants. The other group of important genes identified in our study includes *Ca_14209* (associated with two traits: ciceritol and total sugar contents) and *Ca_27229* (associated with raffinose content), and both genes encode *UDP-glucose dehydrogenase* (*UGD*) and likely interfere with RFOs biosynthesis as a competitor for the upstream precursor compound (Joët et al., 2009).

A candidate gene *Ca_23689* harboring SNP Ca4_43438450 was associated with raffinose content, and GO annotation indicates gene *Ca_23689* encodes structural constituent of the cell wall (**Table 2**). A precedent study has discovered that over-expression of *raffinose synthase* (*rfs*) resulted in increased biomass and total cellulose content in the cell wall (Unda et al., 2017). Gene *Ca_03642* was associated with chickpea stachyose content in seed (**Table 2**). The GO term functional annotation suggests that gene *Ca_03642* involves intracellular protein transport, vesicle-mediated transport, and membrane fusion. Studies have demonstrated that stachyose is the primary photoassimilate and transport sugar in legumes (Peterbauer et al., 2001; Qiu et al., 2015). Another gene, *Ca_09762*, which is predicted as involved in calcium ion transmembrane transport, was also associated with stachyose content (**Table 2**). Meanwhile, we identified another SNP locus (Ca6_2510863) in the gene *Ca_10383* associated with sucrose content in chickpea seed, and the functional prediction of gene *Ca_10383* is ATP hydrolysis coupled proton transport. A previous study suggested that stachyose and sucrose may also be accumulated in the vacuole by stachyose and Sucrose/H^+^ antiporter mechanisms, which is an ATPase energized vacuolar uptake process (Keller, 1992).

Two SNP loci (Ca_46225454 in gene *Ca_21541* and Ca5_1870839 in gene *Ca_26715*) were associated with ciceritol content in chickpea seeds (**Table 2**). Gene *Ca_21541* is predicted as a *TPX2* (targeting protein for Xklp2) family protein. SNP Ca_46225454 is also adjacent to a sequence fragment ortholog of *AT5G40690*, which encodes for methyltransferase activity. Ciceritol is the end product of the inositol methylation process, which explains the role of the identified SNP Ca_46225454 in ciceritol biosynthesis in chickpeas. Gene *Ca_26715* is an *endochitinase A-like protein*. Chitinase is known to protect plants against abiotic and biotic stresses (de Las Mercedes Dana et al., 2006; Karlsson and Stenlid, 2008; Xin et al., 2021; Zhang et al., 2022). The association between endochitinase and ciceritol content in chickpea suggests a metabolic link between the ciceritol pathway and the pathway leading to biotic and abiotic resistance. Two SNPs were identified for total sugar content: SNP Ca4_17208915 in gene *Ca_05402* and Ca5_1870839 in gene *Ca_26715*. Gene *Ca_05402* involves ATP hydrolysis coupled proton transport. Gene *Ca_26715* is predicted as an *A-like endochitinase* (**Table 2**).

## Discussion

### Breeding specialty chickpeas

Identification of germplasm lines with good nutritional quality parameters is key for developing cultivars for different end users. In this study, we have identified varying sugar profile accessions that could be exploited as a base in breeding specialty chickpeas. Especially the accessions with high sucrose (ICC 12564 and ICC 9137) and low RFOs (ICC 6263 and ICC 13816) are desirable for making delicacies like hummus, besan laddoo, Mysore Pak, besan barfi, Puran Poli, and other confectionaries without refined sugars and or artificial sweetening agents. The low RFO lines could be exploited in human and animal food and feed industries. Stachyose and raffinose are considered the most undesirable oligosaccharides present in chickpea. The low stachyose and raffinose accessions ICC 13816 and ICC 6263 can be used in breeding programs as a unique germplasm resource to develop chickpea varieties with improved nutrient utilization and digestibility. The chickpea mini-core collection also shows a considerable amount of variation for ciceritol among accessions. It is believed that these compounds play an important role in protecting plants and seeds against drought stress (Keller and Ludlow, 1993). Ciceritol, a new trisaccharide do not correlate with flatulence, was found high in chickpea accessions reported by Quemener and Brillouet (1983) and Xiaoli et al. (2008). Further research indicated that ciceritol also plays an important role in improving gut health by enhancing the growth of *Lactobacillus, Enterococcus*, and *Bifidobacterium* spp in addition to the production of short-chain fatty acids and is used as a potential source of prebiotics (Zhang et al., 2017). Therefore, increasing ciceritol relatively decreases the flatus potential of chickpea.

### Seed oligosaccharide candidates

There are four genes - two associated with stachyose (*Ca_06204: phosphatidylinositol N-acetylglucosaminyltransferase; Ca_04353: Type I inositol 1,4,5-trisphosphate 5-phosphatase*) and two associated with sucrose (*Ca_20828: inositol-polyphosphate phosphatase; Ca_10185*:*UDP-glucose dehydrogenase*) – have been recognized as indispensably involved in various signaling pathways in plants *via* mediating the phospholipidation and phosphatization of *Myo-inositol* and its derivatives like sucrose and RFOs. Studies reported the crosstalk linkage between inositol signaling and sugar metabolism in plants (Saddhe et al., 2021; Lou et al., 2007; Yang et al., 2007). In 2008, Ananieva et al. (2008) discovered the *in-vitro* interactions between the *myo-inositol polyphosphate 5-phosphatase (5PTase13)* and the *sucrose nonfermenting-1-related kinase (SnRK1.1)* and identified *5PTase13* regulated *SnRK1* activity under different sugar conditions. Plant *SnRK1* is known for its key role at the interface among sugar metabolism, stress signaling, and other physiological developmental processes like seed germination and seedling growth (Radchuk et al., 2006; Baena-González et al., 2007; Jossier et al., 2009; Hulsmans et al., 2016; Elango et al., 2022b). *SnRK1* has been identified in higher plants and two other subfamilies – *SnRK2* and *SnRK3* (Halford and Hey, 2009). Purcell et al. (1998) demonstrated that *SnRK1* plays an essential role in the regulation of *Suc synthase* expression in potatoes (*Solanum tuberosum*), and later Tiessen et al. (2003) recognized that *SnRK1* also regulates starch biosynthesis. The same finding about *SnRK1*’s role involved in starch synthesis was also identified in pollen grains of barley (*Hordeum vulgare*) (Zhang et al., 2001). Our findings indicate that in chickpea, the stachyose and sucrose biosynthesis is mediated by various kinds of *inositol-polyphosphate phosphatase*, potentially through their regulation of *SnRK* families. Additionally, the genes involved in the RFO biosynthetic pathway have been identified in soybean (*Glycine max*) and common bean (*Phaseolus vulgaris*). de Koning et al. (2021) identified three *galactinol synthase* (*GolS*) genes in common bean, named *PvGolS1, PvGolS2*, and *PvGolS3*. *GolS* crosslinks between *inositol* and RFO biosynthesis, and *GolS* are the primary checkpoint for RFO biosynthesis *via inositols* (Sengupta et al., 2015).

We also found that three candidate genes encode for *remorin* protein – two genes (*Ca_17399* and *Ca_22050*) are associated with raffinose content, and the other gene (*Ca_11152*) is linked with total sugar content in chickpea seeds. RFOs serve as a major transport form of carbohydrates in the vascular system in plants (Ayre et al., 2003; Johnson et al., 2020; Ren et al., 2021). *Remorin* is a kind of plant-specific membrane-bound protein and has been identified in *Arabidopsis thaliana*, *Nicotiana tabacum*, *Medicago truncatula*, and *Lycopersicon esculentum* (Watson et al., 2003; Bariola et al., 2004; Marmagne et al., 2004; Mongrand et al., 2004; Sazuka et al., 2004; Nelson et al., 2006; Valot et al., 2006). In Arabidopsis, 16 genes have been identified in the *REM* family, and among them, *REM1.2, REM1.3, and REM1.4* from the *REM1* subfamily were found to exist ubiquitously in the majority of the tissues (Huang et al., 2019). Previous studies demonstrated that *remorin* proteins are localized in the plasma membrane and plasmodesmata of phloem companion cells and regulate photoassimilate translocation *via* reducing plasmodesmata permeability in the symplastic system in rice (*Oryza sativa*) (Gui et al., 2014). As an example, in rice, over-expressed *remorin* gene *gsd1-D* in the dominant mutant (*grain setting defect1-Dominant*) showed a grain setting-deficient phenotype of reduced grain setting rate, reversible accumulation of carbohydrate in leaves, and reduced synthesis of soluble sugar concentration in phloem exudates (Gui et al., 2014).

Three candidate genes are also identified as *UDP-glucose dehydrogenase (UGD)* – gene *Ca_14209* is simultaneously associated with two traits (ciceritol and total sugar contents). The other two genes are *Ca_27229* (associated with raffinose content) and *Ca_10185* (associated with sucrose content). *UGD* is a key enzyme in carbohydrate metabolism and has been identified in soybean (*Glycine max*), maize (*Zea mays* L.), cotton (*Gossypium hirsutum*), and Arabidopsis (*Arabidopsis thaliana*) (Kärkönen et al., 2005; Kohlberger et al., 2018; Jia et al., 2021). The overexpression of UDG-coding gene *PeUGDH4* in Arabidopsis leads to a significant increase in hemicellulose synthesis (Yang et al., 2020), indicating its critical role in plant cell wall synthesis. *UGD* converts UDP-glucose to UDP-glucuronic acid, providing the precursor for hemicellulose and pectin biosynthesis - the two confound components in the primary cell wall matrix (Oka and Jigami, 2006). And later, *UGD* has also been identified to be highly involved in the secondary cell wall construction in Moso bamboo (*Phyllostachys edulis*) (Yang et al., 2020). Besides being incorporated into the cell wall, the remainder forms of carbohydrates can be small molecule oligosaccharides such as RFOs. The RFOs biosynthesis pathways started with UDP-galactose being converted to UDP-galacturonic acid (*UDP-GalA*) by UG4E UDP-glucose 4′-epimerase; or alternatively UDP-glucose being converted to myo-inositol (Karner et al., 2004; Joët et al., 2009). Then both *UDP-GAL* and *myo-inositol* can be the precursors of the galactinol biosynthesis by *galactinol synthase GolS* (Keller and Pharr, 1996). Galactinol is believed to be the only known galactosyl donor to RFOs (Sprenger and Keller, 2000). In summary, the cell wall polysaccharide (CWP) and RFOs biosynthesis pathways are interconnected but also competitive for the upstream precursor UDP-glucose. The increase in UGD activity could lead to the increased production of CWP; however, at the same time, it could diminish RFOs and other carbohydrates production.

## Conclusion

The present study identified potential candidate genes regulating the biosynthesis and transport of seed oligosaccharides in chickpea. We have identified 48 SNPs associated with five sugar types. Nine genes (*Ca_06204, Ca_04353*, and *Ca_20828*: *Phosphatidylinositol N-acetylglucosaminyltransferase*; *Ca_17399* and *Ca_22050*: *Remorin proteins*; *Ca_11152*: *Protein-serine/threonine phosphatase; Ca_10185, Ca_14209*, and *Ca_27229*: *UDP-glucose dehydrogenase*) were identified as potential candidate genes for sugar metabolism and transport in chickpea. The accessions with low RFOs and high sucrose contents may be utilized in breeding specialty chickpeas. The identified candidates could be exploited in marker-assisted breeding, genomic selection, and genetic engineering to improve the sugar profiles in legumes and other crop species.

## Data availability statement

The original contributions presented in the study are included in the article/**Supplementary Materials**. Further inquiries can be directed to the corresponding author.

## Author contributions

DE- designed experiments, collected and analyzed data, and wrote the manuscript. WW- analyzed data and wrote the manuscript. MT- analyzed the data. SS- designed experiments, collected and analyzed data. BR- analyzed and wrote the manuscript. RV- edited the manuscript. All authors contributed to the article and approved the submitted version.

## Funding

The open access publication fees for this article were covered by the Iowa State University Library.

## Conflict of interest

The authors declare that the research was conducted in the absence of any commercial or financial relationships that could be construed as a potential conflict of interest.

## Publisher’s note

All claims expressed in this article are solely those of the authors and do not necessarily represent those of their affiliated organizations, or those of the publisher, the editors and the reviewers. Any product that may be evaluated in this article, or claim that may be made by its manufacturer, is not guaranteed or endorsed by the publisher.
